# Factors influencing the implementation of a school-based parental support programme to promote health-related behaviours—interviews with teachers and parents

**DOI:** 10.1186/s12889-015-1896-x

**Published:** 2015-06-09

**Authors:** Helena Bergström, Ulrika Haggård, Åsa Norman, Elinor Sundblom, Liselotte Schäfer Elinder, Gisela Nyberg

**Affiliations:** Centre for Epidemiology and Community Medicine, Stockholm County Council, Box 1497, 171 29 Solna, Sweden; Department of Public Health Sciences, Karolinska Institutet, Tomtebodavägen 18A, 171 77 Stockholm, Sweden; National Board of Forensic Medicine, Box 4044, Huddinge, Sweden

**Keywords:** A healthy school start, Health promotion, Barriers, Diet, Facilitators, Implementation, Parental support, Physical activity, School children

## Abstract

**Background:**

The ‘Healthy School Start’ programme was developed to promote healthy dietary habits and physical activity, targeting parents of 6-year-old children in pre-school class. Knowledge of barriers and facilitators of implementation is crucial before introducing this kind of programme on a larger scale. The aim of this study was to explore the views of teachers and parents regarding factors influencing the implementation of a school-based parental support programme to promote physical activity and healthy diet.

**Methods:**

An inductive qualitative method was used to explore the experiences and views of teachers and parents involved in the programme. A group discussion was held with three teachers, and semi-structured interviews were conducted with 14 parents. Data were analysed using qualitative content analysis.

**Results:**

Clear communication on roles and responsibilities was identified as an overarching theme, emphasising the importance of clear information and well-functioning cooperation between project management, schools and parents when implementing the programme in a school setting. Five categories at a manifest level described aspects influencing the implementation: 1) ‘The programme’ underlining the importance of flexibility and feed-back; 2) ‘the school’ referring to management and work routines; 3) ‘family conditions’, implying various life situations; 4) ‘group dynamics’ dealing with attitudes among children and parents; and 5) ‘the surrounding community’ including accessibility and attitudes within society.

**Conclusions:**

When implementing a parental support programme in a school setting it is important to facilitate communication and clearly define the division of responsibilities between project management, schools and parents. This emphasises the need for managerial support, and a professional prevention support system.

## Background

Health among Swedish children is in general good, with a relatively high proportion of children with high self-rated health and a low proportion of obesity, compared to other European countries [1–3]. The home environment [4–6] as well as parental styles and practices [6, 7] play an important role in the development of children’s dietary and physical activity habits, and therefore the home is a crucial setting to promote healthy behaviours among children. Another important setting is the school, which has the potential to reduce social inequalities in health because it reaches all children. School-based programmes have shown some effectiveness in promoting health-related behaviours as well as preventing overweight and obesity [8, 9], and the literature suggests that even better results may be obtained if parents are involved [10, 11]. Counselling of parents can be an effective way to improve young children’s dietary habits [12], whereas sending home information is not [13, 14].

Based on this knowledge the programme ‘A Healthy School Start’ was developed with the aim to promote healthy dietary habits and physical activity among children through councelling through MI to parents in a school setting [15]. MI was chosen as a counselling method as studies suggest that MI may increase parents’ motivation to improve their children’s health habits [16, 17]. The programme resulted in a significant increase in vegetable intake in the children and the girls in the intervention group had higher total physical activity during the weekends compared to the children in the control group [18].

In general there is a lack of research regarding barriers and facilitators of implementation of specific programmes [19]. This is essential as even effective health promotion programmes will not make an impact if not implemented as intended.

When trying to implement health promotion programmes on a wider scale it is important to prepare the process thoroughly [20]. By studying barriers to implementation it is possible to develop and select strategies to overcome difficulties [21]. Factors identified to affect the implementation process in general, according to a systematic review by Durlak and DuPre [22], include community level factors, provider characteristics, characteristics of the innovation, organizational capacity, and factors related to the prevention support system.

According to this review, ‘community level factors’ describe contextual aspects which can enhance or impede implementation, such as politics, policies and funding. ‘Provider characteristics’ refers to self-efficacy, skill proficiency and perceived needs among the providers. If the providers believe that a programme will produce desired benefits they are more likely to implement it with high fidelity. ‘Characteristics of the innovation’ concerns compatibility with values and goals of the organisation and adaptability of the intervention. A programme is much more likely to be implemented if it fits with the organisation’s existing priorities and if it is modifiable in accordance with local needs. ‘Organisational capacity’ deals with work climate, practices and leadership. Effective leadership within organisations and shared decision-making has been identified as crucial to implementation. Finally ‘factors related to the prevention support system’ includes training and technical assistance to providers. Training is needed to prepare providers for their task and technical assistance refers to the resources offered to providers once the intervention begins [22].

Although there are many effectiveness studies regarding children’s dietary habits and physical activity to date, there is a lack of studies investigating factors of importance for large-scale implementation. The aim of this study was to explore the views of teachers and parents regarding factors influencing the implementation of a school-based parental support programme to promote physical activity and healthy diet.

## Methods

An inductive qualitative method was used to explore the experiences and views of teachers and parents involved in the programme. Qualitative methods are useful for exploring perceived barriers and facilitators of implementation [20] and permit the researcher to study selected issues in depth and detail [23].

### Description of the programme

The programme ‘A Healthy School Start’, described in detail elsewhere [15], was developed for children in pre-school class and their parents. Briefly, the programme consisted of three components; 1) A brochure sent home to each family, 2) Motivational Interviewing (MI) with the parents, and 3) Teacher-led classroom activities with the children. The intervention was carried out in seven pre-school classes with 6-year-old children in a municipality in Stockholm County, Sweden. The control group also consisted of seven pre-school classes in the same area, but was not included in the present study. The duration of the intervention was six months, and the primary outcome was physical activity, assessed by accelerometers. Dietary intake was assessed through parental questionnaires and weight and height of the children was measured.

#### A brochure sent home to each family

The brochure included information about seven different areas: 1) Parental feeding practices, 2) healthy food and family meal times, 3) physical activity, 4) sweets, snacks ice-cream and sodas, 5) fruit and vegetables, 6) physical inactivity, screen time and commercials, and 7) sleep.

#### Motivational Interviewing (MI)

The parents were offered two sessions of MI, 45 min each, approximately three months apart. One hundred and ten parents participated in the first session and 89 parents in the second session. The sessions were conducted by an MI counsellor with documented competence in MI who was also a member of the research team. The sessions were held in the schools and were scheduled in accordance with requests of the parents, often in mornings or late afternoons. During the first session the parents were asked to choose an issue regarding their child’s diet, physical activity or sleep that they wanted to change from an agenda setting tool and had the opportunity to set a goal for the family at the end of the session. During the second session the parents explored their efforts towards achieving that goal or explored further behaviour change targets. Parents were excluded from the second session if they had not participated in the first session.

#### Teacher-led classroom activities with the children

The classroom activities included ten 30-min teacher-led sessions that conveyed “take home” messages to enforce parents. The teachers were provided with a manual for the sessions and a tool-box with pedagogic materials, such as food models made of cardboard and illustrated information on how much fruit and vegetable consumption is recommended. The children received a workbook, which they brought home after each session as homework to conduct together with their parents. The homework could for example be to look for healthy foods in the grocery store, to do a family activity or to try out a new fruit or vegetable and to draw it.

### Implementation strategies

Strategies for implementation targeted the schools, the teachers and the parents. The principal in each school signed a contract with the research group specifying the obligations and commitments of the school and the research team. Before the start of the intervention the teachers were trained for the classroom activities for two hours. The training and supervision of teachers was done by the research team. Parents were informed about the project at the first regular information meeting in school for new parents.

### Setting and participants

The schools where the programme was carried out were located in a municipality with around 100.000 inhabitants. The average income and educational level was relatively high, but there were notable gradients in income and educational level of families in different geographical areas. The schools included in this study were mainly located in areas with low to medium socio-economic status [15].

To obtain different perspectives, and thereby achieve triangulation to support trustworthiness [23], data were collected from teachers as well as from parents. All seven teachers included in the intervention were invited to participate in the study. Parents were chosen by two members of the research team (ES and ÅN) as a purposeful sample with maximum variation [23]. The variation was based on the following variables: sex of parent, sex of child, ethnicity, participation in one or two of the MI-sessions, behaviour targeted in the session/s (diet, physical activity or TV habits) and which school the child attended (Table 1). Initially, 20 parents were invited to participate in this study, of whom seven were men and 13 were women. However, five of the invited parents and four of the invited teachers declined participation due to time constraints, or without further explanation. One parent had to be excluded because the interview was not recorded properly. In total, three teachers and 14 parents were included in the study. All of the teachers were women.

**Table 1:** Description of parents

Parent	Sex of parent	Sex of child	Ethnicity	Education	Number of MI sessions	Behaviour focus in MI-session	School class
1	F	F	Swedish	University	1	Diet	A
2	F	M	Swedish	High School	2	Diet	B
3	F	M	Swedish	University	2	Diet	C
4	F	M	Swedish	High School	2	Diet	A
5	M	F	Swedish	University	2	Physical activity	D
6	F	F	Non-European	High School	2	Physical activity	E
7	F	M	European	University	2	TV habits	A
8	M	F	Non-European	University	2	TV habits	C
9	F	F	Swedish	High School	2	None	A
10	F	F	Swedish	University	1	Diet	E
11	F	F	Swedish	High School	2	Physical activity	B
12	F	M	Swedish	High School	1	Diet	D
13	M	M	European	University	2	Diet	C
14	F	F	Swedish	University	2	Diet	D

### Data collection

A group discussion, inspired by focus group methodology, was chosen in order to capture the attitudes and experiences among the teachers [24]. It was assumed that a group discussion would enrich the data as the teachers would be able to compare and discuss their experiences of barriers and facilitators in the implementation of the programme. The discussion was held in a conference room at the town hall in November 2011 and lasted for 90 min. One of the authors (UH) acted as moderator for the discussion, and another author (HB) took notes. None of them had been involved in intervention delivery. Questions were discussed, by predetermined topics, inspired by factors important to implementation according to Durlak and DuPre [22]; 1) Community level factors, 2) Provider characteristics, 3) Innovation characteristics, 4) Organizational characteristics and 5) Prevention support system. The participants were encouraged to talk to each other rather than addressing the moderator [24].

Individual interviews were chosen to collect data from the parents since this method is useful when the purpose is to access the perspectives of people [23]. The interviews with parents were conducted in the schools attended by their child and lasted from about 25 to 50 min. A semi-structured interview guide was developed, based on the five implementation factors described above [22]. Questions to the parents were for example: “what is your experience taking part in this project?”, “what do you think about the materials?” or “what do you think about the teachers involvement in the project?” The questions to the teachers were more dealing with organisational factors like “what support did you get from the head master?” or “what did you think about the support from the research team?” All of the interviews were performed by one of the authors (UH) and carried out between December 2011 and January 2012. The interviews, as well as the group discussion, were audiotaped and transcribed verbatim.

The study was approved by the Regional Ethical Review Board in Stockholm County, No. 2010/934-31/1. Prior to each interview, the participants were informed about their right to withdraw from the interview and were ensured confidentiality.

### Data analysis

The transcripts were read through several times to obtain a sense of the content. The interviews with parents and the group discussion with the teachers were first analysed separately to detect possible differences between the two groups. As there were no major differences, the analyses were then integrated into a coherent whole. The initial separate analyses were kept to facilitate description of minor differences between the teachers and the parents. Data were analysed inductively using qualitative content analysis according to the procedure described by Graneheim and Lundman [25]. Meaning units, i.e. words, sentences or paragraphs containing aspects related to each other through their content and context, were identified and labelled with codes. The codes were compared based on differences and similarities, and sorted into subcategories and categories. Analysis was performed by HB and discussed continuously with the other authors. The theme, as well as categories and sub-categories, were defined by inter-subjective agreement between the authors, to enhance the trustworthiness of the study [25]. In cases of disagreement, the transcripts were carefully reread until consensus on the categorisation was reached.

## Results

We identified an overarching theme at a latent level; Clear communication on roles and responsibilities, and five descriptive categories at a manifest level: 1) The programme; 2) the school; 3) family conditions; 4) group dynamics; and 5) the surrounding community. An overview of the results is given in Table 2.

**Table 2:** Factors influencing the implementation of a school-based parenting support programme, according to teachers and parents

*Clear communication on roles and responsibilities*				
The programme	The school	Family conditions	Group dynamics	The surrounding community
Simplicity and flexibility	Management	Perceived need	Attitudes among children	Accessibility and availability
Meaningful content	Resources	Life situation	Attitudes among parents	Attitudes in society
Support for behavioural change	Work routines	Engagement and interest		
Information and feed-back	School-parent contact	Ability to change		

### Theme: clear communication on roles and responsibilities

The overarching theme highlights the importance of communication between the project management, schools and parents during the implementation of the programme. It was of importance that both providers and participants knew from the start who was responsible for which part of the programme and what was expected from each and every one. Although the information given about the programme was perceived as clear there was a need for additional information. Both teachers and parents were positive towards the programme, but there were some unfulfilled expectations. For example, the parents expected more to happen in school, while teachers expected the parents to get more involved. According to this, clear communication and feedback is important to strengthen and motivate both teachers and parents in their efforts to promote healthy behaviours among children.

### The programme

The programme was perceived as flexible and easy to implement. Parents described homework as non-burdensome and appreciated the flexible scheduling for the MI-sessions. The teachers particularly expressed appreciation for the ready-to-use material, which did not become an additional burden, but rather facilitated their daily work.

*Just that everything was served to you, it was simply amazing. In this way you would do just as much as you wanted yourself, using these materials with books and everything. / Teacher*

The programme was perceived as meaningful, dealing with important issues as well as including challenging tasks for children. Being part of a research project was mentioned as a facilitator. Both teachers and parents perceived that they received support from the programme for behaviour change in several ways. Professionalism was stressed, as well as the educational materials and personal contact with the research team. Not all parents interviewed felt that they had received support through the MI-sessions, but several parents expressed that these sessions were the most important part of the programme, helping them to articulate what changes they wanted to do and in which way.

*When I wrote my goals, and then there was someone else listening to my goals. It was helpful; I’d still … disclose my problems. The interviewer, who I knew had listened to what I said … and believed that the goals were good. And then I felt some pressure to follow them up as well. / Parent 3*

The importance of the face-to-face communication was emphasized, although some parents discussed the possibility of support via telephone or email. The teachers would have liked more time to reflect upon their work together with their colleagues and some parents asked for more concrete ideas on how to solve everyday issues. According to the teachers, it was important that the responsibility for the programme and its implementation was clearly defined. If the programme were not managed by a research team they thought that someone working in the municipality should take the lead. Although both teachers and parents stated that they received clear information about the programme, several parents requested more frequent feedback. They especially asked for feedback on the study results, both at an individual and at a group level. Some parents described how reminders about the programme would facilitate their motivation to engage in the programme, giving them the feeling that their contribution was important.

### The school

According to the teachers, the school needed support from outside to manage the programme. Within school they asked for more interdisciplinary cooperation. Teachers emphasised that the school’s resources are limited and that the workload is high. The classroom component was perceived to be well adapted to the school setting, and easy to integrate into daily routines. Parents primarily perceived the programme as a school project, and several of them expressed a wish that more action would have taken place in the school to support the children. Both teachers and parents stressed the importance of good cooperation between the school and the parents. The parents felt that they received too little information, while some of the teachers felt they provided information to the parents without getting any response.

*We sent out weekly newsletters every week. What we kind of wrote and, yes, the homework of the week and so on… We also reminded the parents, but it did not work that well after all. / Teacher*

*I just really did not know, had no follow-up from school, what … what had happened or what they were doing. Yes, because then we would have been able to be more supportive at home. If you know that this week we aim for this. Yes, fine, then … then we continue with it at home as well. / Parent 4*

### Family conditions

The perceived need for support regarding diet and physical activity varied among the families. Some described difficulties regarding vegetable consumption and watching TV, while others felt that their habits already were satisfactory. Even parents who did not experience needs for support appreciated the idea of paying attention to the children’s habits. The life situation of the parents, in terms of work and family constellation, affected their ability to get involved in the programme. Among divorced parents the information did not always reach both parents. Several parents also described a stressful life with a heavy workload, having more than one child and various leisure activities for them. Parents’ interest in the programme varied, while teachers stated that it was very important to involve the parents. Involving the children was also described as important, but not as difficult. Some parents described a strong self-efficacy in influencing the children’s lifestyles, while others felt more uncertain about their parental authority.

*We’re pretty … very clear with our children. So they know what the rules are. And then we … we should not say that we are strict, but they know that when I say no then it’s no, then it’s not nagging twenty times. / Parent 9*

*You can change a little bit, but I’m not strong enough to say no, no candy. I cannot … I’m weaker. / Parent 7*

### Group dynamics

The children were influenced by attitudes among their peers, for example whether other children disliked vegetables or whether it was considered important to follow certain TV series.

*And it’s not possible to turn it off either, because then it becomes … ‘why can’t I watch when my friends do’ and … Maybe they are talking about what happens in this series in school then. And the one who does not watch, he’s … he knows nothing (laughs). / Parent 8*

The parents’ attitudes towards the programme and health behaviours were affected by other parents’ attitudes. Some parents mentioned that other parents’ rules mattered and others called for parental cooperation to agree on common rules.

### The surrounding community

Some of the parents mentioned factors in the environment affecting their ability to support their children towards healthy habits, such as climate, weather, food prices, exposure to healthy and less healthy foods, and accessibility to sport activities.

*Because it is a problem today that perhaps children and even we, the adults, are more sedentary and we have been overly exposed to fast food. / Parent 1*

Attitudes and focus in the media and in society were also perceived to have an impact. One of the parents discussed the impact of the food industry and the teachers mentioned that the health topic is “hot”.

## Discussion

In this study we have explored the views of parents and teachers regarding the implementation of a school-based parental support programme targeting children’s dietary habits and physical activity. Our results show that there are a number of barriers which could potentially hinder successful implementation of the programme, as well as facilitators supporting the implementation process.

The results imply that clear communication between the research team, schools and parents is of major importance when implementing such programme in a school setting, as well as each and everyone’s expectations on their own and other people’s responsibilities. This conclusion is supported by earlier research regarding the implementation of alcohol and drug programmes in schools, which emphasises the importance of clear and mutually agreed upon definitions and expectations when schools are to be engaged [26].

The programme was perceived as easy to implement by the teachers, because it included ready-to-use material and both the contents and the time scheduling of the classroom component was flexible. School programmes which are simple to conduct and do not involve too extensive changes from the current practice are known to be easier to implement [26]. Already in 1995 Rogers pointed out that for effective implementation of a new programme this should be no more complex than existing services [27]. This becomes a challenge, because such a programme might not be comprehensive enough to be effective [28]. In this case the programme was perceived as relatively simple, because the teachers were thoroughly guided through each step of those routines although it did include new routines. However, it should be remembered that the person conducting the MI sessions was a member of the research team in this study, and mobilising this capacity in school might well turn out to be a barrier in the implementation of this programme, due to the need for extra resources.

Two programme characteristics that have been related to implementation are ‘compatibility’ , which refers to the extent to which the intervention fits with the priorities and values of the organisation and ‘adaptability’ , which refers to the extent to which the programme can be adapted to local needs [22]. Programmes that are congruent with existing norms are more likely to be successfully implemented [22]. School programmes also need to be designed with enough flexibility to have local relevance [19]. This programme was perceived as contextually appropriate while at the same time being flexible regarding time and content, which might have been especially important because the workload in schools was described as high. The MI-sessions for the parents allowed for individual tailoring, which has been pointed out as an important facilitator for parental engagement in parenting programmes [28].

The programme was perceived as meaningful, and even more so because it was a research project. This aspect has to be considered, because it implies that this kind of programme might be viewed as less important when scaled up and implemented without support from a research team. To maintain the perception of importance it might be necessary to give continuous feedback on the progress made, which points to the need for external leadership. The importance of personal contact, especially regarding MI-sessions, was emphasised by some of the parents. Telephone-based interventions can have effect on children’s dietary habits [29–31], but according to some parents in this study support via telephone or email would lack an important dimension.

According to the teachers the classroom component fitted well into existing school routines and was easy to implement. This indicated a high feasibility and possibilities for successful integration of this component in the school setting [32]. It became clear that if it was not run by a research team, someone else would have to provide external support for the programme. Effective leadership is crucial to implementation [22] and lack of leadership support has been identified as a barrier to implementation for other school-based programmes [33]. Our findings suggest that it is essential to designate a project leader within the municipality, such as a health coordinator.

The importance of good cooperation between the school and the parents was stressed, but there were obvious challenges relating to the dissemination of information. This kind of difficulty in school-parent communication has been described previously [34]. As communication and clarity in the division of responsibilities is important, this underlines the need to find well-functioning communication channels. In a qualitative study from four countries parents expressed a preference for informal personal contact above written communication through letters, websites and diaries [35].

Perceived needs and family conditions affected the parents’ motivation and ability to engage in the programme. This category relates to ‘provider characteristics’ [22], although parents could be viewed both as providers and target group in this programme. Providers who believe that a programme will produce desired benefits, feel confident in their ability to do what is expected and, being proficient, are more likely to implement the programme as intended [22]. This would be applicable to both teachers and parents. A challenging family situation might be a barrier though. Competing demands on parent’s time and resources has been described as a barrier for engagement [28] as also described by the parents during the MI sessions [36].

Group dynamics among the children and the parents affected the attitudes towards the programme as well as towards behaviour change. Some parents called for parental cooperation, which could be one way of strengthening the parental engagement. Because some of the parents experienced high stress as a barrier to be as engaged as they would like to, they might have hoped for parental cooperation as a way to reduce this stress. Previous research confirms that high work-life stress has a negative impact on parents’ possibilities to create an environment that stimulates healthy dietary and physical activity habits [36–39].

The surrounding community, including the social as well as the physical environment, affected the families and their perceived possibilities to change behaviour. Factors in the home and in the school environment have shown strong correlations with physical activity and dietary habits in children [6, 40]. To be able to create this supportive home environment the parents might be supported or hindered by attitudes in society as well as accessibility to a supportive environment or lack thereof. Other community level factors, mentioned by Durlak and DuPre, regard politics, funding and policy [22]. These factors were not taken up by parents or teachers but are nevertheless important to consider while planning further implementation.

### Strengths and weaknesses

This study contributes valuable knowledge when implementing a parental support programme in a school setting. Data was collected from both teachers and parents, which increases the credibility of the study [23]. Illustrative citations and intersubjective agreement in the coding and analysis of the data increases the trustworthiness as well [23]. The setting and the intervention are described in detail and therefore the results should be transferable to similar programmes and contexts. It is a limitation that the the perspective of the children was not covered, but would be useful to investigate in future studies. Because the MI-sessions were delivered by the research team it was not possible to realistically evaluate the implementation of this component, which might be seen as a limitation. In the future we hope to be able to integrate the programme into school health care services, which means that school nurses or other staff will be responsible for the delivery of the MI. A relatively small number of informants were included, but the teachers represented different schools and the parents were purposefully sampled and therefore represented a wide variation in experiences. The relatively small number of teachers participating may however limit the transferability of the study.

## Conclusion

When implementing a parental support programme in a school setting it is important to facilitate communication between project management, schools and parents. This emphasises the need for managerial support, and a prevention support system. The implementation can be facilitated by clear information to everyone involved prior to start, a flexible programme allowing some adaptation to local needs, a programme content perceived as meaningful, face-to-face communication and support characterised by professionalism. To further facilitate motivation among teachers and parents, results should be reported back, together with other relevant reminders. Barriers found included limited resources and high workload within the schools, stressed family situations, and lack of support within the physical and social environment. Future research should identify the best way to provide MI-sessions for parents when the programme is implemented on a wider scale.

## Abbreviation

MI: Motivational interviewing
